# Should Root Plasticity Be a Crop Breeding Target?

**DOI:** 10.3389/fpls.2020.00546

**Published:** 2020-05-15

**Authors:** Hannah M. Schneider, Jonathan P. Lynch

**Affiliations:** Department of Plant Science, The Pennsylvania State University, University Park, PA, United States

**Keywords:** anatomy, architecture, breeding, crop, ideotype, plasticity, root

## Abstract

Root phenotypic plasticity has been proposed as a target for the development of more productive crops in variable environments. However, the plasticity of root anatomical and architectural responses to environmental cues is highly complex, and the consequences of these responses for plant fitness are poorly understood. We propose that root phenotypic plasticity may be beneficial in natural or low-input systems in which the availability of soil resources is spatiotemporally dynamic. Crop ancestors and landraces were selected with multiple stresses, competition, significant root loss and heterogenous resource distribution which favored plasticity in response to resource availability. However, in high-input agroecosystems, the value of phenotypic plasticity is unclear, since human management has removed many of these constraints to root function. Further research is needed to understand the fitness landscape of plastic responses including understanding the value of plasticity in different environments, environmental signals that induce plastic responses, and the genetic architecture of plasticity before it is widely adopted in breeding programs. Phenotypic plasticity has many potential ecological, and physiological benefits, but its costs and adaptive value in high-input agricultural systems is poorly understood and merits further research.

## Introduction

Unpredictable growth environments, decreasing freshwater availability, altered precipitation patterns, ongoing soil degradation, and the rising cost of nitrogen and phosphorus fertilizer demand the development of crop varieties that are resilient to abiotic stress (Tebaldi and Lobell, 2008; Brisson et al., 2010; Woods et al., 2010; Sandhu et al., 2016; Lynch, 2019). Root phenotypic plasticity is a widespread and important phenomenon for the optimized capture of edaphic resources. An array of biotic and abiotic constraints limit plant productivity, and phenotypic plasticity is an important phenomenon to enable plants to adapt to spatiotemporal changes in their environment. In this article we consider the benefits and tradeoffs of root phenotypic plasticity in the development of more productive annual agricultural crops. Many studies of phenotypic plasticity measure the plastic response of allometric traits (length, volume, or biomass), which display plasticity, but may not be adaptive, as they merely reflect growth itself. Many ecological studies of phenotypic plasticity focus on comparisons of distinct species, which is not as relevant to crop improvement as comparisons of genotypes within a species. We will not attempt to provide a comprehensive review of a large and disparate literature, much of which only has tangential relevance to annual crops, but instead focus on opportunities and costs of plasticity for root anatomical and architectural phenotypes in agroecosystems.

**FIGURE 1:**
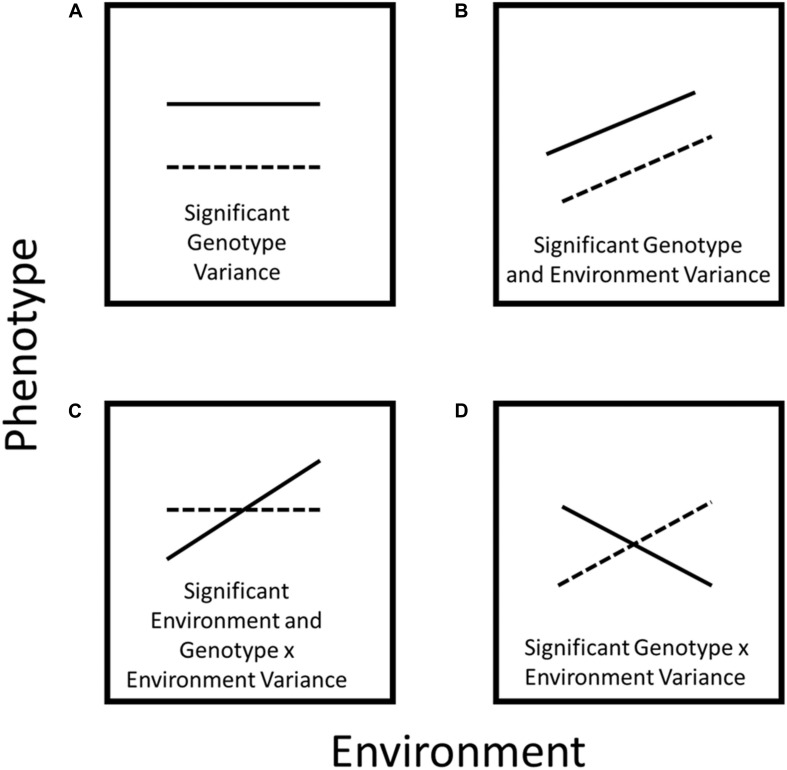
Schematic diagram of plastic responses. In **(A)** the phene value does not change across environments, however, phene expression varies between genotypes. In **(B)** the phene value changes across environments but the reaction norm runs parallel because the response to the environment is the same for both genotypes. In **(C)** one genotype does not exhibit plasticity for a specific phene, while another genotype demonstrates significant environmental plasticity. In **(D)** the reaction norms cross because there is a strong plastic phenotypic response to different environments for both genotypes.

The classic paradigm is that a phenotype (P) is the product of genetics or intrinsic developmental processes (G), environment (E), and the interaction between genetics and the environment (G × E) (Sultan, 2000). Phenotypic plasticity is the ability of an organism to alter its phenotype in response to the environment and may involve changes in physiology, morphology, anatomy, development, or resource allocation (Figure 1; Sultan, 2000). Plasticity is not a characteristic of an organism as a whole, but rather is a characteristic of a given phene (“phene” is to “phenotype” as “gene” is to “genotype”) (Lynch, 2011; Pieruschka and Poorter, 2012; York et al., 2013) in response to a given environment. A phene state is the outcome of complex synergistic developmental systems, influenced by many genes and gene products, as well as the environment (Miklos and Rubin, 1996; Trewavas and Malho, 1997). Plastic responses can affect the fitness of a genotype and be a response to physical, chemical, and biological processes or resource limitations (Weiner, 2004). The phenotypic spectrum, or an array of possible phenotypes a single genotype can display in a single environment, illustrates that many factors influence the expression of a phenotype. For example, the effects of roots of neighboring plants and priority effects determined by germination time may have large effects on the expression of a phenotype in a single environment (Xie et al., 2019). Phenotypic plasticity may include components of genotype by environment interaction, adaptation, and acclimation.

Biologists have long been aware of plasticity (which is one reason that many experiments are performed in controlled environmental conditions), and for much of the past century phenotypic plasticity has been regarded as “noise” and was thought to obstruct the true or native phenotype of an organism. In a paper entitled “The problem of environment and selection,” Falconer argued that environmental effects were a major problem in breeding programs since they interfered with the artificial selection of a trait (Falconer, 1952). However, it is now understood that plasticity is genetically controlled, heritable, and important for the evolution of the species (Bradshaw, 2006). Phenotypic plasticity is now recognized as a significant source of phenotypic variation and diversity and is an important aspect of how organisms develop, function, and evolve (Sultan, 2000).

Phenotypic plasticity may be adaptive, maladaptive, or neutral in regard to fitness. In the heterogenous matrix of soil, many phenes and combinations of phene states may have utility for resource capture and display a wide range of variation, providing opportunity for plastic responses to evolve. Phenotypic plasticity has utility in enabling a genotype to produce better adapted phenotypes and phenotype-environment combinations across more environments than would be otherwise be possible. However, if no tradeoffs or constraints existed, organisms should be able to exhibit perfect or infinite plasticity by expressing the more adaptive phene or combinations of phenes in every environment with no cost. Costly, but maladaptive or neutral phenotypic responses are expected to go extinct (Dewitt et al., 1998) and we would only expect costly forms of plasticity to persist if they have fitness value.

A plastic response does not imply an adaptive response, although many types of plasticity have important adaptive effects. Adaptive plasticity (positively associated with fitness) and apparent plasticity [lacking adaptive value (e.g., specific types of allometry or stress responses); Correa et al., 2019] are both types of plasticity. Maladaptive plasticity can occur when a plastic response that was adaptive in an evolutionary context is counterproductive in a novel environment. This is especially relevant for crop breeding, since many agroecosystems, especially high-input agroecosystems, differ sharply from ancestral selection environments, as discussed below. By definition, allometric responses to the environment may be considered plastic responses, however, they are often just a function of alterations in plant size (or development), and they may not necessarily be adaptive. For example, maize plants with greater biomass had increased stele cross-sectional area and number of metaxylem vessels, which is not necessarily an adaptive response (Yang et al., 2019). However, changes in allometric partitioning (e.g., changes in root to shoot partitioning) may be adaptive by refocusing plant resources to address resource shortfalls (Bloom et al., 1985). In order to interpret differences in biomass allocation, it is necessary to distinguish these sources of variation. It is difficult to distinguish apparent plasticity from plasticity that may be adaptive.

By definition, edaphic stress reduces plant growth, which is a plastic response but is not necessarily adaptive. For example, reduced grain yield or total root biomass under drought is not an adaptive response, but is a plastic response to stress (Ehdaie et al., 2012) and different growing environments and/or different genotypes may display different rates or types of developmental retardation in response to the same stress. In contrast, the plastic response of genotypes during stress recovery may be adaptive. Phenotypic plasticity encompasses a wide range of environmental responses.

Here we focus on understanding the fitness landscape (i.e., how phenes affect crop performance in an array of environments and phene combinations) of root anatomical and architectural phenotypes in agroecosystems. We discuss the benefits and trade-offs to plasticity and the utility of root plasticity in monocots and dicots, acid soils, high and low input environments, and polycultures. We also review the genetic architecture and potential breeding strategies of root phene plasticity. Additionally, we highlight future research directions for root plasticity to enable a comprehensive understanding of the fitness landscape and integration into breeding programs.

## Root Phenes Are Important for Resource Capture

Root phenes have important roles in soil resource capture, especially in environments with suboptimal water and nutrient availability. Root anatomical and architectural phenes determine the temporal and spatial distribution of root foraging in specific soil domains and hence the capture of mobile and immobile resources (Lynch, 1995, 2013, 2019; Hirel et al., 2007; Lynch and Brown, 2012; Lynch and Wojciechowski, 2015). Mobile soil resources, including nitrate and water, are generally more available in deeper soil domains over time due to crop uptake, evaporation, and leaching throughout the growth season. In contrast, immobile soil nutrients, including phosphorus and potassium, are more available in the topsoil (Lynch and Brown, 2001; Lynch and Wojciechowski, 2015). Plants that are able to acquire edaphic resources at reduced metabolic cost will have increased productivity and performance by permitting greater resource allocation to growth, continued soil resource acquisition, and reproduction (Lynch, 2013, 2015, 2018, 2019). For example, root growth angle influences root depth, and therefore plant performance in nutrient and water stress conditions (Bonser et al., 1996; Uga et al., 2011; Trachsel et al., 2013; York et al., 2013; Dathe et al., 2016) since steep growth angles enable deeper rooting and the capture of mobile nutrients in deep soil domains (Trachsel et al., 2013; Dathe et al., 2016) while shallow growth angles are more beneficial for the capture of immobile resources in the topsoil (Bonser et al., 1996; Lynch and Brown, 2001; Ho et al., 2005; Zhu et al., 2005c).

Root anatomical phenes improve plant growth and performance in edaphic stress by reducing the nutrient and carbon costs of tissue construction and maintenance (Lynch, 2013, 2015, 2018, 2019). Root cortical aerenchyma are air-filled lacunae that result from programmed cell death in root cortical cells (Drew et al., 2000). Air-filled lacunae replace living cortical parenchyma, thereby reducing root segment respiration and nutrient demand (Saengwilai et al., 2014a; Chimungu et al., 2015; Galindo-Castañeda et al., 2018). The reduction in tissue maintenance costs associated with the formation of root cortical aerenchyma enable roots to explore deeper soil domains and improve the capture of water and nitrogen, and thereby improve plant growth and yield in environments with low water and nitrogen availability (Zhu et al., 2010a; Jaramillo et al., 2013; Saengwilai et al., 2014a; Lynch, 2015; Chimungu et al., 2015). Similar to root cortical aerenchyma, a reduction in the number of cortical cell files or an increase in cortical cell size also results in a reduction in tissue maintenance and/or construction costs which enables deeper rooting and improved plant growth in drought environments (Chimungu et al., 2014a, b). In temperate small grains, root cortical senescence enables greater exploration of deeper soil domains and greater plant growth in edaphic stress due to reduced cortical burden (Schneider et al., 2017a, b; Schneider and Lynch, 2018). In common bean, reduced secondary growth resulted in reduced specific root respiration and subsequently greater shoot mass and root length in phosphorus-stress conditions (Strock et al., 2018). Plastic responses of root phenes may have large implications in the capture of edaphic resources.

In the field, plants may be exposed to successive or multiple, simultaneous stresses. For example, in conditions of terminal drought, seeds are planted in moist soil but the soil progressively dries from the surface due to drainage, evaporation, and plant water uptake, resulting in relatively greater water availability in deeper soil strata and progressively harder topsoils in most agroecosystems (Lynch, 2013; Lynch et al., 2014). Root tissue construction and maintenance demand significant resources, and in bean cumulative tissue maintenance demands may exceed root tissue construction costs after 1 week of growth (Nielsen et al., 1994, 2001). The investment of those carbon and nutrient resources in tissue construction and maintenance early in plant growth limits the opportunity for the construction of additional roots in different soil domains as resource availability changes. For example, if roots proliferate early in the growth season in the moist topsoil, this limits the opportunity for the construction of roots in deeper soil domains where resources are likely to be located later in the growth season. In addition, early root proliferation in topsoil may not have utility in hard, dry soils later in the season. Root deployment therefore implies opportunity costs, especially during multiple successive or simultaneous stresses.

Root architectural and anatomical phenes have important roles in the capture of soil resources in specific environments, for example sustained nitrogen or phosphorus stress (Lynch, 2013, 2018, 2019), however, root phene states can be functionally maladaptive in fluctuating environments or environments with multiple simultaneous stresses (Ho et al., 2005; Poot and Lambers, 2008). For example, shallow growth angles can improve topsoil foraging and improve the capture of phosphorus, but may be functionally maladaptive for the capture of deep resources like water (Ho et al., 2005). In common bean, shallow growth angle and greater number of basal root whorls and hypocotyl-borne roots increase total root length in the topsoil resulting in greater phosphorus acquisition (Rangarajan et al., 2018). However, as the number of axial roots and/or basal root whorl number increase, the resulting carbon limitation leads to a reduced root depth and therefore trade-offs for the capture of deep resources, such as nitrogen (Rangarajan et al., 2018). In monocots, in which axial roots emerge from shoot nodes, shallow roots lack the ability to forage for deep resources, while deep rooting permits the capture of deep resources like nitrogen and water while also being capable of capturing shallow resources, thus creating asymmetric phenotypic trade-offs for the capture of deep and shallow resources (Lynch, 2013). No single phene state is optimal across a range of environments and management practices (Dathe et al., 2016; Tardieu, 2018; Rangarajan et al., 2018).

## Many Root Phenes Are Plastic

**FIGURE 2:**
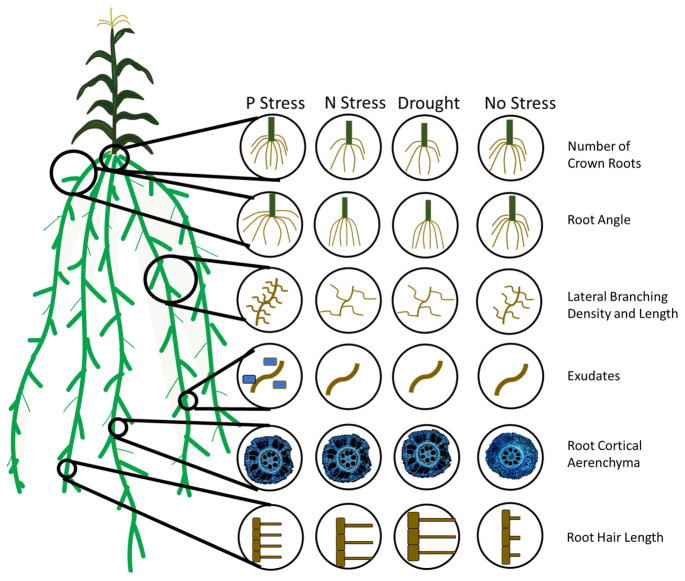
Adaptive root phene plasticity to optimize soil resource capture in edaphic stress. A number of root phenes have been demonstrated to have an adaptive plastic response to edaphic stress. In phosphorus stress, plants with many nodal roots with a steep angle, many short lateral branches, root exudation, root cortical aerenchyma formation, and long, dense root hairs are adaptive or proposed adaptive responses for stress tolerance. In nitrogen and water stress, few crown roots with a steep angle, few long lateral branches, root cortical aerenchyma formation, and long root hairs are adaptive or proposed adaptive responses for stress tolerance.

**FIGURE 3:**
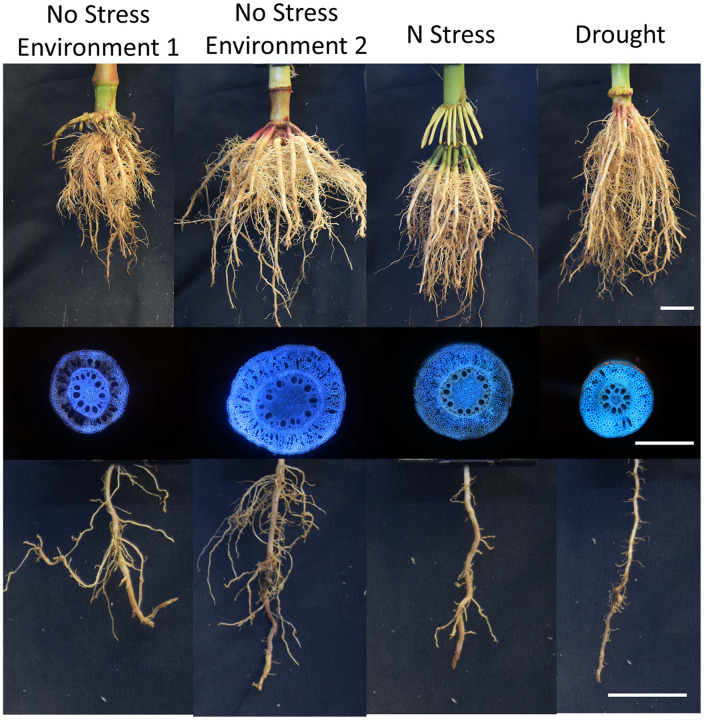
Gentoypes vary in their plastic response to environment, nitrogen stress, and drought. Architectural and anatomical images are presented from a single genotype in response to different environments and edaphic stress conditions. Phenotypic plasticity is shown for root architecture, root anatomy, and lateral branching length and density. Scale bar represents 2 cm (root crown and lateral branch) and 1 mm (anatomy).

**FIGURE 4:**
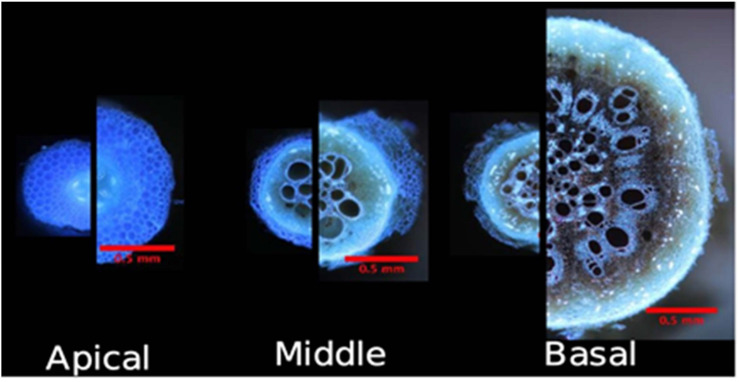
Secondary root growth in common bean (Phaseolus vulgaris) is plastic in response to phosphorus availability. Comparison of basal root anatomy under high P and P stress in greenhouse conditions at 46 DAP. A11 cross-sections are at the same scale. Modified and reproduced with permission from Strock et al. (2018).

**FIGURE 5:**
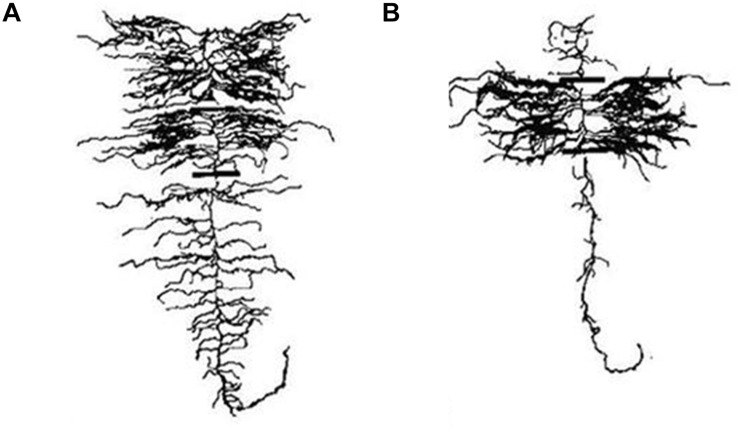
Lateral root proliferation of barley in response to a nutrient patch. **(A)** A plant supplied with a uniform treatment of nitrate has a uniform lateral branching density and length along the axial root. **(B)** A plant supplied with nitrate through a banded treatment displays lateral root proliferation in the banded region. Modified and reproduced with permission from Drew (1975).

**FIGURE 6:**
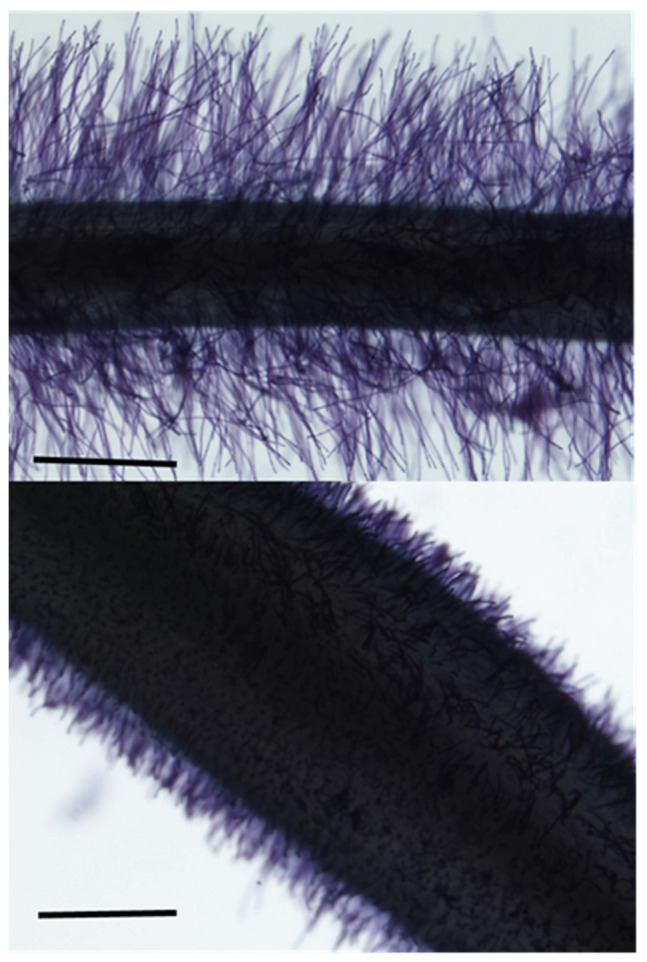
Root hair length is plastic in response to 10W phosphorus environments. Root hairs become longer in low phosphorus environments and are associated with greater shoot biomass. Root hair image courtesy of Anica Massas.

Plasticity has been observed for a number of root anatomical and architectural phenes (Figures 2, 3). In soybean grown under drought, metaxylem vessel number increased, thereby improving root hydraulic conductivity, while reducing total cortical area which reduced the metabolic cost of accessing water in deep soil domains (Prince et al., 2017). In drought and low phosphorus environments, increased plasticity of root architecture traits correlated with high yield stability in rice (Sandhu et al., 2016). In water stress, plasticity in root length and root cortical aerenchyma formation has been observed in rice and was associated with greater shoot biomass and yield (Niones et al., 2012, 2013). In water stress in wheat and rice, xylem vessel diameter and number and stele diameter were highly plastic (Kadam et al., 2017). Greater phenotypic plasticity in wheat root anatomical traits may be associated with greater stress tolerance compared to rice (Kadam et al., 2017). In common bean, plasticity in secondary root growth influenced root depth and shoot growth in low phosphorus environments (Figure 4; Strock et al., 2018). In rice, plasticity in lateral root length and density (Kano et al., 2011; Kano-Nakata et al., 2013), root length density, and total root length (Kano-Nakata et al., 2011; Tran et al., 2014) correlated with greater shoot biomass, water uptake, and photosynthesis in drought. The number of nodal roots in rice (Suralta et al., 2010) and maize (Gao and Lynch, 2016), lateral branching density and length in maize (Zhan et al., 2015), and deep rooting in wheat (Ehdaie et al., 2012; Wasson et al., 2012), millet (Rostamza et al., 2013), rice (Hazman and Brown, 2018), and maize (Nakamoto, 1993) also have displayed plastic responses to water deficit. A plastic response of lateral root proliferation was induced in barley in response to patches of nitrogen (Figure 5; Drew et al., 1975) and in maize in response to phosphorus patches (Yano and Kume, 2005). Hydropatterning is a plastic response involving the development of lateral branches, root hairs, and aerenchyma toward available water (Bao et al., 2014). Maize genotypes with plastic root hairs that became longer under low phosphorus had better performance under low phosphorus availability than genotypes with constitutively long root hairs (Figure 6; Zhu et al., 2010b). Root anatomical and architectural phenes express a wide range of plastic responses in a wide range of environments. However, it is unclear which plastic responses are adaptive and how phenes interact to create adaptive responses to edaphic stress.

## Potential Benefits and Tradeoffs of Phenotypic Plasticity

There are many examples of adaptive plasticity of root phenes, including the increased development of root cortical aerenchyma, fewer lateral root branches in water deficit, or deeper distribution of lateral root branches, and it has been proposed that phenotypic plasticity may be the future of crop breeding since it would enable the development of more efficient crops that could adapt to changing environments (Gifford et al., 2013; Hazman and Brown, 2018; Lobet et al., 2019). Adaptive plasticity may promote establishment and persistence in novel environments and allows genotypes to have broader tolerance and greater fitness across environments. It has been proposed that understanding the genetic and mechanistic basis of root phenotypic plasticity will enable the rapid development of more productive crop varieties that will be robust and stable in future climates (Topp, 2016). The adaptation of taxa to sudden environmental changes, like those caused by human disturbance, could also be an advantage of plasticity since these changes generally occur at too rapid of a pace for an evolutionary response, or the development of new crop cultivars through breeding.

However, “perfect” plasticity is unattainable due to an inability to consistently produce the optimum phenotype, fluctuating environmental signals, and/or because phenotypic plasticity comes at a cost (León, 1993; Via and Lande, 2006). A cost of plasticity is when a plastic organism exhibits less fitness while producing the same phene state as a fixed organism. Costs of plasticity have been identified in a variety of systems (Relyea, 2002; Merilä et al., 2004). Maintenance cost of phenotypic plasticity may be incurred if facultative development requires the maintenance or construction of sensory and regulatory machinery that fixed development does not require.

Genetic costs of plasticity also exist. Phenotypic plasticity may manifest because structural genes or their products are directly affected by the external environment (i.e., allelic sensitivity) or because regulatory genes are affected by the environment which in turn affect the expression of structural genes (Via et al., 1995). However, genetic linkage may cause genes associated with plasticity to be linked with genes conferring reduced fitness, plasticity genes may have negative pleiotropic effects on phenes other than the plastic phene, or epistasis may cause the regulatory loci producing the plastic response to modify expression of other genes. With little known about the molecular mechanisms and genetic control of the plastic response, linkage and pleiotropic effects could severely limit the productivity of plastic crop varieties.

In specific environmental scenarios, plasticity may limit plant productivity. For example, if environmental information is not reliable, plastic organisms can produce maladapted phenotypes when environmental cues are incorrectly interpreted, or when correct signals are interpreted about the initial environment, but the environment fluctuates or is highly variable. In many cases, especially with developmental or morphological plasticity, the development of tissues takes time and often there is a lag time between environmental cues and the development of tissues expressing the plastic response. For example, nitrogen, phosphorus, and water are all growth regulators but have different mobilities in soil. Nitrogen and water are mobile and can move faster through the soil profile than plants are able to respond by constructing new tissues or modifying established tissues. Even in the case of phosphorus, an immobile soil resource, changes in phosphate uptake kinetics contribute more to increased phosphorus acquisition than root proliferation in heterogeneous soil environments (Jackson et al., 1990; Caldwell et al., 1992). It also has been suggested that genotypes with fixed development (i.e., non-plastic phenes) may be able to express more extreme phene states than plastic genotypes since there may be a trade-off between the developmental range that can be expressed across habitats and the magnitude of expression within an environment (Wilson and Yoshimura, 1994; DeWitt, 1998).

Costly, but maladaptive or neutral phenotypic responses are expected to go extinct (Dewitt et al., 1998) and we would only expect costly forms of plasticity to persist if they have fitness value in some seasons or environments. We speculate that plasticity was a useful mechanism for crop ancestors to grow and develop in novel environments and thrive in unmanaged, unfertilized, and non-irrigated natural ecosystems. In low-input systems, plasticity may be advantageous by exploiting resource patches with increased lateral root proliferation which may confer a competitive advantage (Lynch, 2018). However, in modern agricultural environments with high-inputs, plasticity may come at a greater cost than a benefit. Indirect evidence for this is the observation that during selection of modern temperate maize breeding, regions of the genome contributing to G × E variance and plasticity were not directly or indirectly selected to increase plant productivity and yield stability (Gage et al., 2017).

Short duration plasticity, or physiological plasticity, in variable environments may be advantageous in specific environments, however, plasticity may be maladaptive in high-input environments with intensive fertilization and greater nutrient availability. In high-input environments, constraints for soil resource acquisition and plant growth in stress are mitigated and strategies that evolved in environments with biotic and abiotic stress influencing root function may not have utility in these high-input environments (Lynch, 2018). Root phenotypes that explore deep soil domains, whether plastic or not, enhance the capture of deep resources like water and nitrogen in most agricultural systems, despite the fact that water and nitrogen availability are sometimes greater in surface soils of high-input systems (Manschadi et al., 2006; Gowda et al., 2011; Henry et al., 2011).

If a population is exposed to a novel environment and becomes successful, but becomes restricted to that environment, alleles that contributed to plastic responses in the new environment should trend toward fixation in the absence of gene flow from other populations and therefore their ability to confer plasticity is also reduced (Mitchell-Olds et al., 2007; Anderson et al., 2017; Gage et al., 2017). The utility of phenotypic plasticity in successful and highly productive modern crop varieties in heavily managed high-input environments is limited and not required for the survival or migration of the species.

## Plasticity in the Context of Temporal Resource Duration

The expression of plant phenes as a result of plasticity may be of variable duration and plastic responses may be long- or short-term. Short-term plasticity, is also referred to as physiological plasticity, allows plants to adjust to temporally variable aspects of the environment such as water or nitrogen availability. For example, the expression of aquaporins or nitrate transporters fluctuates as a short-term response to water or nitrogen availability (Feng et al., 2011; Zargar et al., 2017). In contrast, changes due to morphological or developmental plasticity may be of longer duration (Sultan, 2000). For example, the size and number of cortical cells or initial root angle is established near the growing root apex, and potential for change in mature tissues is limited. Phenotypic plasticity that is established early in development, such as root growth angle, may be beneficial in conditions of sustained edaphic stress (e.g., low phosphorus availability), but may be maladaptive in stresses that fluctuate on shorter time scales (e.g., drought, low nitrogen availability) by creating sustained responses to ephemeral conditions (Lynch, 2013). In addition, the timing of development itself, and its response to the environment, may be plastic. Developmental plasticity may be limited to early growth stages, or its timing may vary in different genotypes or species (Pigliucci and Schlichting, 1995). For example, the development of root cortical senescence has the greatest utility in edaphic stress conditions when development occurs relatively early in plant growth, however, genotypic contrasts exist for the rate and timing of its development in root cortical tissues (Schneider et al., 2017b).

In response to heterogeneous soil conditions, root plasticity can also vary spatially. Lateral root branches have been documented in some species and genotypes to proliferate in response to localized patches of nutrient availability (Figure 5; Drew, 1975; Zhu and Lynch, 2004). Lateral root proliferation in response to nutrient patches has been proposed as a beneficial strategy for enhanced nitrogen acquisition (Mi et al., 2010), however, if mobile resources move faster through the soil profile than roots can proliferate, this response may be maladaptive. In some species, plasticity of lateral root branching in response to local nutrient patches may enhance nutrient resource capture in environments with sustained nutrient sources or in conditions of interspecific competition (Robinson et al., 1999). However, this can be detrimental when proliferation in response to local nutrients diverts resources from other soil domains with greater resource availability, particularly deeper soil domains in leaching precipitation regimes later in the growing season (Lynch, 2013, 2018).

## Utility of Root Plasticity Varies Between Dicots and Monocots

Monocots and dicots have different foraging strategies for edaphic resources. Throughout the growth season, monocots continually produce new roots from stem nodes, and tillers. In contrast, new roots of dicots are predominately lateral roots arising from older root axes. Dicots do have younger hypocotyl-borne roots that emerge throughout the growth season, however, they normally do not comprise a large portion of the root system, which usually consists of relatively few axial roots of larger diameter with a highly developed lateral root system having multiple orders of lateral branching. Monocots may have superior topsoil foraging, as new flushes of roots are continuously pushed down through shallow soils, whereas in dicots many new roots form in deeper soil domains (Lynch, 2013). In addition, in tillering monocot species, an optimum number of tillers should exist to enhance capture of edaphic resources as the number of tillers is directly related to the number of adventitious roots (Hecht et al., 2016). Reduced crown root number improves plant growth with low nitrogen (Saengwilai et al., 2014b) and drought (Gao and Lynch, 2016) by reducing inter- and intra-plant competition for internal and external resources, thereby increasing root depth and acquisition of deep soil resources. However, greater crown root number improves plant growth in low phosphorus soil by reducing axial root elongation and improving topsoil foraging (Sun et al., 2018). We speculate that the number of tillers (and therefore the number of adventitious roots originating from tillers), and its plastic response to plant density and stress, is important for edaphic stress tolerance in monocot species.

There are important differences between the anatomy of monocot and dicot roots. Roots of dicot species radially expand through secondary growth, which has important implications for edaphic stress tolerance. Phosphorus stress reduces secondary growth in *Phaseolus vulgaris* in a genotype-dependent manner, and genotypes with greater reduction of secondary growth had reduced metabolic costs, increased root length, improved phosphorus capture, and increased shoot biomass in low phosphorus soil (Figure 4; Strock et al., 2018). In monocots, temperate small grain species develop root cortical senescence (RCS), a type of programmed cell death. Simulation studies suggest that RCS may be an adaptive trait for water and nutrient acquisition. RCS reduces the carbon and nutrient costs of soil exploration by destroying living cortical tissue, thereby reducing carbon and nutrient costs of maintaining a living cortex. The development of RCS may be plastic as limited phosphorus and nitrogen availability accelerate the development of RCS (Schneider et al., 2017a, b). After the development of RCS in monocots or secondary growth in dicots, assimilates that would have been partitioned to the root for maintenance of the cortex may be used for the growth of shoots or new roots, which can increase soil exploration. Monocots and dicots have different foraging and resource acquisition strategies and therefore may have different adaptive plastic responses for soil resource capture.

## Utility of Root Plasticity for Acid Soils

Acid subsoils (generally defined as having a pH < 5) present several challenges to root growth and resource acquisition including aluminum (Al) toxicity, deficiency of phosphorus (P), calcium (Ca), magnesium (Mg) and potassium (K), and possibly manganese (Mn) toxicity. In acid soils, the solubility of Al increases and injury to root apices occurs, therefore reducing root growth, soil exploration, and subsequent resource acquisition.

Commonly, acidic soils are located in humid environments with weathered soils, and acidity increases with soil depth. Plasticity of root phenes that increase topsoil foraging would be beneficial by improving the capture of resources that have greater availability in the topsoil, including P, Ca, Mg, and K (Lynch, 2019), while also avoiding subsoils with greater acidity and Al toxicity (Lynch and Wojciechowski, 2015). The tradeoff of reduced access to deep soil water would probably be less important in humid environments because of greater water availability in shallower soil domains. Topsoil foraging can be improved through a shallower axial root growth angle (Bonser et al., 1996; Liao et al., 2001), greater production of axial roots (Walk et al., 2006; Miguel et al., 2013; Rangarajan et al., 2018; Sun et al., 2018), denser lateral roots (Postma et al., 2014; Jia et al., 2018), and greater root hair length and density (Zhu et al., 2010b; Miguel et al., 2015). Reduced root metabolic cost improves growth in soils with low phosphorus availability. In maize, the formation of root cortical aerenchyma reduces root respiration and the phosphorus cost of maintaining root tissue therefore improving plant growth in low phosphorus (Postma and Lynch, 2011; Galindo-Castañeda et al., 2018). In bean, phosphorus stress inhibits secondary growth of roots which reduces root costs and improves phosphorus capture and plant growth in low phosphorus soils (Strock et al., 2018). Plastic root phenes that improve topsoil foraging may be beneficial for improved capture of phosphorus in acidic soils.

Plasticity in carboxylate exudation may also be an important mechanism for phosphorus uptake in acidic soils. Carboxylate exudation into the rhizosphere solubilizes phosphorus from metal complexes (Ryan et al., 2012). Carboxylates also can precipitate toxic levels of aluminum in the soil (Lambers et al., 2003). Exudation of carboxylates in plant roots is a common phenomenon in many plants including rice (Kirk et al., 1999), wheat (Ryan et al., 1995), and lupin (Gardner et al., 1983). The exudation of carboxylates including citrate and malate into the rhizosphere can incur a large carbon cost (Lambers et al., 2013). Plasticity in the spatiotemporal control of carboxylate exudation, i.e., exudation triggered by aluminum toxicity and phosphorus stress may permit a reduction in the metabolic burden of the root.

Low Ca availability is a major challenge to root growth in acid subsoils (Foy et al., 1969). Differences in cell wall composition may influence tissue Ca requirements and plants with reduced internal Ca requirement therefore may be more productive in acid soils (Lynch and Wojciechowski, 2015). Cortical cell size, file number, and aerenchyma all influence the amount of cell wall material per root volume and therefore affect tissue Ca requirement. Genotypes with reduced pectin content, which has a reduced demand for Ca, may also reduce the Ca requirement of the root (Marschner, 1995). We propose that plasticity of phenes that reduce tissue Ca requirements, like increased cortical cell size, reduced file number, reduced pectin content, and increased aerenchyma formation may be beneficial in acid soils. Crops with a reduced Ca tissue requirement may be able to continue to explore acidic subsoils, despite reduced Ca availability and Al toxicity.

## Root Plasticity in the Context of High and Low Input Environments

It has been proposed that wild crop ancestors and landraces produce more roots than directly needed for the capture of edaphic resources to compensate for root loss from biotic stress, edaphic stress, and competition for soil resources with neighboring plants (Lynch, 2018). We speculate that plasticity was a useful mechanism for crop ancestors in natural ecosystems. Short duration plasticity, or physiological plasticity, in variable environments may be advantageous in specific environments, however, plasticity may be maladaptive in high-input environments with intensive fertilization, greater nutrient availability, and reduced biotic stress. In high-input agroecosystems, parsimonious, non-plastic root phenotypes including e.g., fewer axial roots, reduced density and length of lateral roots, reduced cortical cell file number, and reduction of cortical parenchyma through formation of aerenchyma and senescence may be beneficial by permitting deeper rooting and the capture of deep resources like water and nitrogen (Lynch, 2018). Plastic responses to increase topsoil foraging in response to shallow localization of water and N early in the growth season may optimize resource capture in natural systems or low-input agroecosystems, characterized by intense belowground competition from neighboring plants. However, in high-input monocultures, where immobile resources like P and K are likely to be non-limiting, non-plastic phenotypes would be advantageous since eventually water and N would be localized at depth regardless of early season patterns, and resources lost to neighboring plants would still contribute to stand-level fitness (i.e., yield) in high density monocultures (Lynch, 2018). We propose that in low-input systems, highly plastic root phenotypes with a variable number of axial and lateral roots, variable root growth angle, variable length and density of root hairs, variable formation of root cortical aerenchyma and cortical cell files, would be beneficial for the capture of heterogeneous soil resources in environments with significant root loss due to biotic factors. However, in high-input systems, a sparser root system with fewer axial roots may be more beneficial, since the negative effects of biotic stress is diminished (Lynch, 2018).

## Plasticity in the Context of Polycultures

In many low-input agroecosystems, which traditionally consist of polycultures and generally experience greater weed competition, interplant competition with other species has important implications in plant performance. For example, the maize/bean/squash polyculture used in small-scale subsistence farming has a yield advantage over the average yield of the respective monocultures (Mt. Pleasant and Burt, 2010). Maize, bean, and squash have contrasting root architectures (Postma and Lynch, 2012; Zhang et al., 2014) and differences in root architecture and vertical root distribution result in differences in spatial niches and allows polycultures to be productive when plants are competing for soil resources (Zhang et al., 2014). In these polyculture systems, species co-optimize, and spatial niches allow a yield advantage by reducing competition for edaphic resources. In polyculture or multiline systems, highly plastic root architectural phenes could disrupt complementary spatial niche foraging strategies (Zhang et al., 2014). If these species had highly plastic root architectural phenotypes, this would create more competition for the same soil resources, which would be detrimental. For example, if roots of all species proliferate in response to localized patches of nutrient availability, this creates greater inter-plant and species competition. In this scenario, phenotypic plasticity may not be adaptive, as complementary spatial niches are needed for the success of all species or the population.

## Progeny May Be Primed for a Plastic Response

Plants cannot only respond to environmental signals by adjusting their own phenotypes, but also can influence the phenotypes of their offspring, through changes in the quantity and quality of seed production and the structure and quality of the seed coat and fruit tissues (Sultan, 2000). The phenotype of offspring can be influenced by the parental environment. For example, plants can respond to specific environments by changing the structure of thickness of the seed coat while maintaining the quantity and quality of the embryo and endosperm tissues (Sultan, 1996; Lacey et al., 1997). Genotypes may vary in the extent to which seedling and mature root phenes are affected by parental stress. For example, progeny of some common bean genotypes from drought-stressed parents developed fewer and shorter basal roots with smaller diameters (Lorts et al., 2019). Progeny from some genotypes from phosphorus stress parents developed fewer shoot-borne roots and had a greater basal root whorl number (Lorts et al., 2019). Progeny of nutrient-deprived plants increase allocation to root biomass compared to progeny of plants with ample nutrients (Wulff and Bazzaz, 1992). Offspring of light-deprived plants reduce root elongation relative to shoot growth compared to progeny of plants grown in high light (Sultan, 2000). In addition, epigenetic processes, including DNA methylation and histone modification, may alter gene expression and therefore may be important drivers in phenotypic plasticity (Chinnusamy and Zhu, 2009; Nicotra et al., 2010). These plastic changes may enable offspring to maintain critical aspects of plant growth and function, even if the initial seedling biomass is reduced by parental stress.

## Genetic Architecture of Plasticity and Breeding Strategies

Some genetic loci have been associated with root phenes including root stele and xylem vessel diameter in rice (Uga et al., 2008, 2010), xylem vessel phenes in wheat (Sharma et al., 2010), root cortical aerenchyma in *Zea* species (Mano et al., 2006, 2007), areas of cross section, stele, cortex, aerenchyma, and cortical cells, root cortical aerenchyma, cortical cell file number, and length, number, and diameter of nodal roots in maize (Burton et al., 2014a, b). However, genes associated with phene expression are distinct from those associated with plasticity for that expression. Genes associated with plasticity have been identified for root hair length (Zhu et al., 2005a) and lateral root branching and length (Zhu et al., 2005b) in low phosphorus availability in maize, root length density and root dry weight (Sandhu et al., 2016) in rice in response to drought, lateral root branching in rice in response to fluctuating moisture levels (Niones et al., 2015), and wheat and rice root anatomical phenes in response to drought (Kadam et al., 2017). In maize, genes associated with plasticity in response to water deficit and different environments are distinct for cortical phenes, root angle, and lateral branching phenes (Schneider et al., 2020a, b; Table 1). Understanding the genetic architecture of plasticity could provide useful breeding targets for crop improvement in specific environments and improve our understanding of phenotypic plasticity. Plasticity is heritable, and this enables selection for or against plasticity in manmade populations (Pigliucci, 2005). Historically, breeding programs have focused on selecting crop varieties based on uniformity and yield stability in specific environments and management practices, and plasticity has often considered to be a breeding obstacle (Basford and Cooper, 1998; Cooper et al., 1999). Large and complex genotype by environment interactions complicate the design and implementation of breeding strategies (Cooper et al., 1999) and breeders often select for a low genotype by environment contribution to enable genotypes to perform predictably in specific environments. Crop breeding has made huge advancements in the development of productive varieties that are stable across diverse conditions and recent studies have suggested that plasticity was not directly or indirectly selected for in the development of modern crop varieties (Gage et al., 2017). It is important to note that maladaptive plasticity in a specific environment may be adaptive in different environments, including future climates. Plasticity that is not currently adaptive can provide sources of variation that may be important for phenotypic evolution or variation for breeding (Lande, 2009).

**TABLE 1:** Summary of identified genetic loci associated with root plasticity and architecture.

Species	Root trait	Response	References
Soybean	Root length, Number of adventitious roots, Number of root tips	Waterlogging	Ye et al., 2018
Rice	Root diameter, Stele diameter, Cortical diameter, Metaxylem vessel number and diameter, Root length, Specific root length, Root volume, Root surface area	Drought	Kadam et al., 2017
Rice	Lateral root branching	Drought	Niones et al., 2015
Rice	Root length density and root dry weight	Drought	Sandhu et al., 2016
Arabidopsis	Root volume, Weight, Deep root weight	Drought	Li et al., 2017
Arabidopsis	Root length and dry weight	Drought	El-Soda et al., 2015
Maize	Lateral root branching and length	Low Phosphorus	Zhu et al., 2005b
Maize	Root hair length	Low Phosphorus	Zhu et al., 2005a
Maize	Root cortical aerenchyma, Cortical cell size and file number, Metaxylem vessel area, Cortical area, Stele area, Root cross-sectional area	Drought	Schneider et al., 2020a
Maize	Root angle, Lateral root branching length and density, Distance to the first lateral branch	Drought	Schneider et al., 2020b

*Several genes have been identified in many different species for a number of anatomical and architectural phenes.*

The genetic architecture of plasticity is highly complex and quantitative. Many genes with small effects control plastic responses and distinct genes control plastic responses of different root phenes and in response to different stresses and environments (Schneider et al., 2020a, b). This can pose a challenge for breeding programs that use conventional tools like single-trait breeding strategies and marker assisted selection, as hundreds of genes would need to be stacked for the development of desirable root ideotypes for specific environments. However, modern breeding methods, like genomic selection enable the selection of multiple loci.

In addition, genes controlling root anatomical and architectural phenes and their plastic responses are probably highly pleiotropic. For example, multiple root anatomical and architectural phenes are regulated by ethylene (Takahashi et al., 2015; Schneider et al., 2018). Ethylene signaling induces root cortical aerenchyma and RCS formation via programmed cell death (Evans, 2003; Schneider et al., 2018) and presumably common signaling pathways (e.g., ethylene) control expression of other root phenes under a range of edaphic stresses [i.e., lateral root formation (Negi et al., 2008)]. For example, the upregulation of an ethylene-related gene may be intended to increase aerenchyma formation for adaptation in drought environments, however, increased ethylene production may also have unintended effects such as reduced axial root elongation which may be maladaptive in these environments. We must fully understand the genetic architecture of phene plasticity as well as the function of phenes and phene aggregates in order to develop adaptive crop cultivars for specific environment.

In breeding programs with capabilities to use genomic selection, selection should include phenes and integrated phenotypes (and their plastic responses), not just selection for yield. Selection for individual phenes has merits compared with brute-force yield selection for edaphic stress (Lynch, 2019). In training sets for genomic selection, consideration must be given to wild germplasm and landraces, since elite germplasm has been developed through selection in high-input environments and often against plasticity. Landraces and wild germplasm presumably express more phenotypic plasticity than uniform, stable elite crop germplasm and could provide unique sources of phenotypic variation.

Phenotypic selection for plasticity may also be a viable strategy for breeding programs, however, selection must occur in specific targeted environments or under specific edaphic stresses. A genotype that displays adaptive plastic responses to water stress may not express an adaptive (or any) plastic responses to other edaphic stresses such as limited nutrient availability. The phenotyping of plasticity should be evaluated for individual phenes, as plasticity in a variety of phenes and phene combinations can result in similar yield or measures of plant performance.

The adaptive value of plasticity in breeding programs is limited by distinct genetically controlled plasticity responses to different environmental conditions. Breeders may need to target a specific plastic response of a specific phene or set of phenes to a specific abiotic or biotic stress or environment, rather than just breed for a variety that highly expresses phenotypic plasticity. Genotypes that have a plastic response to water deficit are not the same set of genotypes with a plastic response to different environments (i.e., G × E) (Schneider et al., 2020a, b). Breeding efforts to develop varieties that are plastic to a wide range of environments and stresses, may be maladaptive in environments with multiple stresses or stresses that fluctuate on short time scales or that vary throughout the growth season. The development of new crop varieties can take decades, and the utility of phene states in the current target environment may change in future environments and climates. Since each plastic response to an environmental cue has distinct genetics, use of plasticity as a selection criterion is challenging for breeders who must target each plastic response to a specific environment or stress.

## Future Research Directions

Should root plasticity be a breeding target? The answer is complex. The fitness landscape of root phenotypic plasticity is dependent on specific agroecologies and management practices, and the genetic control of plasticity is in general highly quantitative and is dependent on many loci having small effects. To better understand and interpret plasticity, first we need a comprehensive understanding of the utility of individual phenes. Numerous studies evaluate plasticity of specific root length, root biomass, or yield. However, specific root length may depend on the expression of many individual phenes including the formation of root cortical aerenchyma, cortical cell file number, and stele area. Previous studies have demonstrated that phenotypic plasticity is phene-specific, not necessarily genotype-specific (Schneider et al., 2020a, b) so it is important to measure individual phenes as opposed to phene aggregates. When plasticity of a phene aggregate, or combinations of multiple elemental phenes, is measured, it may reflect a plastic response of one or multiple phenes. In addition, when phene aggregates are measured, phenotypic plasticity may be masked by different responses of elemental phenes. For example, the diameter of the root may not exhibit plasticity, but the stele size, cortical cell file number, or size of cortical cells may have changed their phenotype. Many combinations of elemental phenes have the potential to produce the same expression of combinations of phenes.

Contrary to earlier neo-Darwinian views of plasticity as trivial “noise,” plasticity is now considered to be an important source of phenotypic variation. Root systems consist of multiple phenes, each under distinct genetic control, that interact with each other and the environment to determine fitness. The fitness landscape of root phenes and their plastic responses that vary among genotypes, species, and environment is poorly understood. Plants are not equipped with unlimited phenotypic plasticity, which suggests that there are constraints to its expression (Schlichting, 1986).

Several recent studies have focused on the utility of specific phenes in edaphic stress (Trachsel et al., 2013; Chimungu et al., 2014a, b; Saengwilai et al., 2014a, b; Schneider et al., 2017a; Strock et al., 2018), however, the utility of many other root phenes in edaphic stress remains to be explored. In addition, recent studies have explored interactions between root phenes which may be synergistic or antagonistic in nature (Miguel et al., 2015; Rangarajan et al., 2018). For example, in dicots tradeoffs exist between shallow and deep soil foraging (Ho et al., 2005). Recent studies suggest that plasticity is phene-specific and a single genotype may produce an adaptive plastic response for one phene and maladaptive plastic response for a different phene on the same plant (Schneider et al., 2020a). Presumably, genes controlling adaptive phenotypic plasticity would have to be stacked in breeding programs to create a suite of adaptive synergistic phenes. Understanding the utility of root phenes and their interactions will have important implications in understanding adaptive or maladaptive plasticity under specific edaphic stresses. In many cases, more detailed and refined phenotyping methods are needed to be able to characterize and phenotype phene states, rather than phene aggregates. In many knockout collections, the annotation of “no visible phenotype” is common and is partly due to the lack of capacity for the plant science community to analyze subtle and complex elemental phenes. Field phenotyping is a bottleneck in crop breeding programs and high-throughput, industrial-scale phenotyping often does not allow for the identification and understanding of subtle, complex phene states. In the context of plant roots, there are many combinations of phenes that affect fitness of a plant in a specific environment. In order to interpret the adaptive value, utility of phenotypic plasticity, and consider plasticity in breeding programs we must first understand the fitness landscape of individual phenes and phene combinations.

To understand patterns of plasticity, we need to better understand and monitor local environments and changes in the environment. Subtle changes in the environment, such as localized nutrient patches, may induce a phenotypic response and if the environment is not carefully monitored, it makes interpretation of the plastic responses challenging (Schneider et al., 2020a). Field environments are often heterogeneous and difficult to monitor and replicate. *In silico* approaches enable the evaluation of many environment and phenotype combinations including those that do not exist in nature (Dunbabin et al., 2013). The use of modern *in silico* approaches will be necessary to understand the complex interactions of the root fitness landscape that are not possible empirically.

Growth differences between controlled and field environments are often overlooked. Planting density, light, temperature, and other growing conditions have large effects on plant growth and are often dramatically different in the field compared to controlled environments and phenotypic correlations between lab and field data are often poor (Poorter et al., 2016). Controlled environments and growing systems do not represent the heterogeneous matrix of the soil and therefore are difficult to use to discover true plant responses. There is a clear need to employ abiotic conditions that are overall more similar to those which the plants experience in the field (e.g., more natural soils, appropriate planting densities, light intensity). Many previous studies on phenotypic plasticity have focused on environmental responses in straightforward traits including biomass and root-shoot ratios (Bradshaw and Hardwick, 1989) and numerous studies have observed plastic or genotype by environment responses of below- and above-ground plant phenes (Robinson et al., 1999; Gage et al., 2017; Rabbi et al., 2017). Now that we have a basic understanding of plasticity, we can move to understanding more complex and subtle aspects of phenotypic plasticity. Single-factor experiments have been important in understanding plastic responses, however, more realistic environmental complexity is needed in studies (e.g., multiple, simultaneous dynamic stresses). For example, understanding plastic responses to multiple constraints is important. Very few studies have tested plastic responses to multiple simultaneous abiotic and biotic stresses.

Short-term or dynamic plasticity is an important but poorly understood component of plasticity that includes the rate of phenotypic response or patterns of development. Plasticity of short duration may be important in maintaining fitness, particularly in fluctuating environments. Dynamic plasticity is challenging to measure, as it requires phenes to be measured over time in many individuals in different environments. Common phenotyping tools require destructive harvests at fixed times or at fixed growth stages and are slow and costly. However, this is critical to understanding plasticity, as the determination of whether plasticity is adaptive or maladaptive depends strongly on its temporal expression.

The extent of variation in expression of plasticity still remains to be explored in many root phenes. Phenotypic plasticity may be an important source of genetic variation to be exploited for the development of crop varieties for future environments. However, breeding for genotypes with plastic responses will be complicated by their complex genetic architecture, genetic and metabolic costs of plasticity, and potential maladaptive responses in many environments.

We propose that some of the main ideas discussed here regarding root phenotypic plasticity are applicable to shoot phenotypic plasticity. In high-input environments, shoot architecture and anatomy is optimized for enhanced plant performance. Similar to root plasticity, we speculate that in high-input environments, shoot plasticity may not be advantageous, since human management has removed many constraints to shoot function. However, shoot phenotypic plasticity ideotypes, benefits, and trade-offs in many ways are not equivalent to root plasticity as soil resources are spatially and temporally dynamic and much more complex than above-ground environments. Like root phenes, we must fully understand the fitness landscape of shoot phenotypic plasticity before its integration into breeding programs.

To harness the power and knowledge of genomic information and agricultural application of plasticity, we need to be able to comprehensively link genetic information to “real world” phenotypes in “real world” environments. We need to measure the adaptive significance of patterns in plasticity and understand the complex pathways that lead from environmental cues to a plastic response. The fitness landscape of plasticity is highly complex, yet poorly understood and merits further research to understand the utility of plasticity for edaphic stress tolerance. The study of phenotypic plasticity involves many disciplines including ecology, physiology, development morphology, genetics, *in silico* biology and evolution and offers many research opportunities to understand links among these areas.

## Author Contributions

JL conceived the idea. JL and HS contributed to the writing of this manuscript.

## Conflict of Interest

The authors declare that the research was conducted in the absence of any commercial or financial relationships that could be construed as a potential conflict of interest.
